# Are “cool” executive function impairments more salient in ADHD symptoms than in reading disability?

**DOI:** 10.1590/1980-57642020dn14-010008

**Published:** 2020

**Authors:** Gabriella Koltermann, Natália Becker, Júlia Beatriz Lopes-Silva, Mariuche Rodrigues de Almeida Gomides, Giulia Moreira Paiva, Vitor Geraldi Haase, Jerusa Fumagalli de Salles

**Affiliations:** 1 Universidade Federal do Rio Grande do Sul, Porto Alegre, RS, Brasil.; 2 Universidade Federal de Minas Gerais, Belo Horizonte, MG, Brazil.

**Keywords:** attention deficit hyperactivity disorder, reading disability, cognition, child, neuropsychology, transtorno do déficit de atenção com hiperatividade, dificuldade de leitura, cognição, criança, neuropsicologia

## Abstract

**Introduction:**

Reading disability (RD) and Attention Deficit Hyperactivity Disorder (ADHD) symptoms often co-occur in school-age children.

**Methods:**

The present study evaluated the performance of 216 Brazilian children from 3^rd^ and 4^th^ grades on “cool” executive function (EF) abilities and phonological processing. The children were divided into three groups: those with ADHD symptoms only, those with RD only, and controls.

**Results:**

MANOVA analyses, controlling for age and nonverbal intelligence, showed worse performance for the RD group, compared to the ADHD symptoms group, on measures of phonological processing (phonemic awareness, phonological short-term memory, and lexical access) and “cool” EF components (orthographic verbal fluency and processing speed). The ADHD symptoms group did not differ from the control group on the majority of the “cool” EF tasks. Compared to the control group, the ADHD symptoms group and the RD group both showed significantly more errors in rapid automatized naming of figures, which evaluates the inhibition component of EF; performance on this task was similar for these groups.

**Conclusion:**

We conclude that children with RD have greater impairment in phonological processing and “cool” EF compared to those with ADHD symptoms. Furthermore, deficits in inhibitory control may be shared among children with both conditions.

## RESUMO

**Introdução:**

Dificuldades de leitura (DL) e sintomas do Transtorno de Déficit de Atenção e Hiperatividade (TDAH) frequentemente coocorrem em crianças escolares.

**Métodos:**

O presente estudo comparou o desempenho em FE “frias” e processamento fonológico de 216 crianças brasileiras de 3ª e 4ª anos, que foram divididas em três grupos: apenas com sintomas de TDAH, apenas em DL e controles.

**Resultados:**

As análises de MANOVA, controlando para idade e inteligência não-verbal, indicaram que o grupo com DL apresentou desempenho significativamente inferior ao grupo com sintomas de TDAH nas medidas de processamento fonológico (consciência fonológica, memória verbal de curto prazo e acesso lexical) e em componentes das FE “frias” (fluência verbal ortográfica e velocidade de processamento). O grupo com sintomas de TDAH não se diferiram do grupo controle na maior parte das tarefas de FE “frias”. Ambos os grupos com sintomas de TDAH e DL apresentaram desempenhos significativamente menores (mais erros) em comparação às crianças de desenvolvimento típico na tarefa de Nomeação Seriada Rápida de figuras que avalia o componente de controle inibitório, e o desempenho foi semelhante entre os grupos.

**Conclusão:**

Conclui-se que crianças com DL apresentam maior comprometimento em processamento fonológico e FE “frias” em comparação àquelas com sintomas de TDAH e que déficits no controle inibitório podem ser compartilhados entre crianças com ambas as condições.

Reading Disability (RD) and Attention Deficit Hyperactivity Disorder (ADHD) are two of the most common neurodevelopmental disorders in children.^1^ It is estimated that between 6% and 17% of school-age children are diagnosed with RD,^2^ and around 5% exhibit ADHD.^3^ Furthermore, the rate of comorbidity for these two conditions is high, at between 15% and 30%. Both conditions are persistent and constitute risk factors for difficulties in academic and social skills.^4^ The multiple deficit model of neurodevelopmental disorders^5^ proposes that ADHD and RD have a multifactorial etiology and that a single disorder is produced by a combination of specific and shared deficits, with the shared deficits accounting for the comorbidity between disorders.^1^^,^ 4

In line with this assumption, most authors view RD as a developmental impairment that is best studied based on cognitive causal models.^6^^,^ 7 RD is a well-known disorder that profoundly disrupts reading development, at the basis of which are phonological processing deficits.^8^ Studies have shown that deficits in other neurocognitive domains, such as Executive Functions (EF), might also be present in children with RD.^9^ A meta-analysis has found an effect size of .67 for differences between children with and without RD in EF.^6^ However, this does not imply that EF are causally related to word reading ability.^10^ Conversely, ADHD is regarded as a more heterogeneous condition that is associated with distinct patterns of neuropsychological impairments.^11^

One important cognitive model of EF distinguishes between “hot” and “cool” EF components.^12^^,^ 13 Both refer to an individual’s ability to function adequately, regulating thought and behavior according to a means-ends logic.^13^ The term “hot” EF denotes the more emotionally loaded aspects of self-regulation, for example, those pertaining to decision making, reward, and motivation, and coordinated by ventral areas of cortico-subcortical prefrontal circuits.^14^ On the other hand, “cool” EF components are less emotionally loaded and are associated with decontextualized thought processes, coordinated by dorsal cortico-subcortical prefrontal networks. This aspect of EF is mostly associated with the parieto-frontal circuit of general intelligence.^15^

The literature consistently suggests an association between “cool” EF impairments and ADHD symptoms.^16^ A meta-analytic^17^ study has shown that EF impairments are associated with ADHD in general, with larger effect sizes for inhibitory control. However, the cited study also reported that individual differences in “cool” EF did not fully explain the variance in ADHD symptoms.

Following the assumptions of the multiple deficit model, neuropsychological studies have sought to identify specific deficits and shared deficits between RD and ADHD. These studies compared groups with RD-only, ADHD-only, comorbid profiles, and typically developing children.^18^^-^ 20 Even when ADHD symptoms were measured with a questionnaire answered by parents and teachers, distinct cognitive profiles were found between children and adolescents with ADHD only and those with RD only.^21^

Purvis and Tannock^19^ compared the performance of children aged from 7 to 11 years (age range similar to that investigated in the current study) in phonological processing and inhibitory control. They observed significant impairments in all phonological processing measures in the RD-only group, compared to the control group and the ADHD-only group. Additionally, the ADHD-only and RD-only groups performed poorly in inhibitory control. Another study^22^ showed that both ADHD and RD are associated with difficulties in inhibitory control, and these difficulties were found to be more severe in RD than in ADHD. Inhibition deficits were marginally present in children (8-12 years old) with ADHD, whereas inhibition deficits were clear in RD, compared to controls without these developmental disorders.^22^

Since ADHD and RD are two of the most common neurodevelopmental disorders, and considering their high comorbidity and negative impact on children’s lives, it is important to analyze and specify the neuropsychological profiles associated with those conditions, as suggested by the multiple deficit model of neurodevelopmental disorders.^5^

Thus, this study aimed to compare performance in “cool” EF and phonological processing between ADHD-only children, RD-only children, and typically developing children. We expected to find the following results: 1) higher impairment in general “cool” EF and phonological processing among RD children compared to children with ADHD symptoms and to controls and, specifically^9^^,^ 23 ^-^ 25; 2) a shared deficit in inhibitory control by children with ADHD symptoms and RD relative to controls.^19^^,^ 22

## METHODS

### Participants

The sample consisted of 216 children, 55.1% girls, aged between 8 and 11 years (*M* = 8.94, *SD* = .71), from the 3^rd^ (*n* = 62) and 4^th^ grades (*n* = 154). All children attended public elementary schools in Porto Alegre (Rio Grande do Sul; *n* = 66) and Belo Horizonte (Minas Gerais; *n* = 150) (Table 1). The exclusion criteria were: uncorrected auditory or visual impairments (reported by parents/guardians); nonverbal reasoning score below the 15^th^ percentile, measured by the Raven’s Colored Progressive Matrices (RCPM);^26^ and comorbidity between ADHD symptoms and RD (n = 6).

**Table 1:** Characteristics of the three groups according to sociodemographic variables, percentile in the RCPM, MTA-SNAP-IV questionnaire score, and LPI percentile (N = 216).

	Controls (N = 159)	ADHD symptoms (N = 20)	RD (N = 37)	F	p
Sex (%) M/F	46.5% / 53.5%	50% / 50%	35.1% / 64.9%	1.80^I^	.40
Age (M ± SD)	8.96 ± 0,70	8.70 ± .65	9.03 ± .76	1.45	.23
School grade (%) 3º/4º	27% / 73%^a^	55% / 45% ^a^	21.6% / 78.4%^b^	7.87 ^I^	.01*
City (%) BH/POA	69.2% / 30.8%	80% / 20%	64.9% / 35.1%	1.42 ^I^	.49
Schools' IDEB^1^ (M ± SD)	6.67 ± .55	6.65 ± .82	6.57 ± .90	.30	.73
RCPM Percentile (M ± SD)	82.79 ± 14.70 ^a^	82.40 ± 16.87^a,b^	73.81 ± 20.89 ^b^	4.71	.01*
Inattention score (M ± SD)	5.88 ± 3.97 ^a^	14.68 ± 4.86^b^	6.19 ± 4.12^a^	39.74	< .001**
Hyperactivity score (M ± SD)	5.66 ± 3.83 ^a^	15.40 ± 4.41 ^b^	5.08 ± 4.32^a^	55.96	< .001**
Combined inattention and hyperactivity/impulsivity score (M ± SD)	11.54 ± 6.74 ^a^	29.74 ± 6.37 ^b^	11.27 ± 6.74 ^b^	63.99	< .001**
LPI percentile (M ± SD)	52.86 ± 27.80 ^a^	48.50 ± 21.58 ^a^	7.04 ± 3.23 ^b^	51.36	< .001**

ADHD: attention deficit hyperactivity disorder. RD: reading disability. M: male. F: female. BH: Belo Horizonte. POA: Porto Alegre. RCPM: Raven Colored Progressive Matrices. LPI: Oral Single-Word and Pseudoword Reading Task. + F: analysis of variance. +^a,b^: Bonferroni post-hoc analysis. § ^I^: Chi-square test. || ^1^= Indicator of school quality in Brazil. This index is calculated based on a combination of school progression rates and performance on government mandatory standardized tests, such as the Brazil Exam and the SAEB. When results were last published, in 2013, the national IDEB for Brazil was calculated at 5.2, well below the value of 6.0 recommended by the Organization for Economic Cooperation and Development.^26^

* p = .02.

** p < .001.

### Study design and procedures

This study was approved by the Research Ethics Committee of the Federal University of Minas Gerais (protocol number 939.562) and the Ethics Committee of the Institute of Psychology at the Federal University of Rio Grande do Sul (protocol number 1.023.371). Two evaluation sessions (a group session and an individual session) were conducted with the children whose parents/guardians signed the Informed Consent Form. The research aims and procedures were explained to the children and parents. Assessments were conducted only after the child’s written and oral informed consent and the parent’s written informed consent were obtained.

The RCPM^26^ was administered in a group session, while the remaining instruments were administered individually (approximately 90 minutes). Parents/guardians answered a questionnaire about sociodemographic/health conditions for each child, as well as the MTA-SNAP-IV.^27^ All instruments were administered by trained researchers, and the same procedures were followed in both Porto Alegre and Belo Horizonte.

This study has a quasi-experimental design of contrasting groups.^28^ The following three groups were formed according to the children’s’ scores on the MTA-SNAP-IV questionnaire,^27^ answered by parents/guardians, and on the Oral Single-Word and Pseudoword Reading Task (LPI)^29^ (Table 1):

a) Inattention/hyperactivity symptoms group (ADHD symptoms group; *n* = 20): comprising children who scored at least 6 items as either “pretty much” or “very much” in questions 1 to 9 (symptoms of inattention) of the MTA-SNAP-IV questionnaire^27^ and/or at least 6 items as either “pretty much” or “very much” in questions 10 to 18 (hyperactivity/impulsivity symptoms), and whose performance was above the 16^th^ percentile on the LPI^29^ (see the Instruments section).
b) RD without ADHD symptoms group (RD group; *n* = 37): comprising children who scored below the cut-off point described above on the MTA-SNAP-IV questionnaire,^27^ and whose performance were below the 16th percentile on the LPI.^29^
c) Control group (without ADHD symptoms or RD; *n* = 159): children who scored below the cut-off point described in section “*a*” of the MTA-SNAP-IV questionnaire,^27^ and with performance above the 16th percentile on the LPI.^29^

### Instruments

The following instruments were employed:

a) Raven’s Colored Progressive Matrices (RCPM):^26^ The age-appropriate Brazilian-validated version of Raven’s Colored Matrices, which assesses nonverbal reasoning.
b) MTA-SNAP-IV:^27^ Abbreviated version of the Swanson, Nolan, and Pelham questionnaire,^30^^,^ 31 which assesses symptoms of inattention, hyperactivity/impulsivity, and oppositional defiant behaviors, answered by parents/guardians or teachers. This instrument was adapted to the Brazilian context.^27^ The questionnaire is composed of 26 items corresponding to the list of symptoms for ADHD and Opposition Defiant Disorder described in the diagnostic criterion A of the DSM-IV, which are virtually the same in the DSM-5. Respondents rated their children’s inattentive (items 1-9), hyperactive-impulsive (items 10-18), and defiant (items 19-26) behaviors using a 4-point Likert scale: 0 (not at all), 1 (just a bit), 2 (pretty much) and 3 (very much).^27^ Moderate to strong correlations were found between the MTA-SNAP-IV and the Brazilian version of the Schedule for Affective Disorders and Schizophrenia for School-Age Children – Present and Lifetime Version (K-SADS-PL). All MTA-SNAP-IV scales showed very high internal consistency coefficients (all above .91).^32^
c) Oral Single-Word and Pseudoword Reading Task (LPI):^29^^,^ 33 This Brazilian oral reading task comprises a set of 60 stimuli selected according to regularity, length, frequency, and lexicality. Stimuli are divided into three categories (20 regular, 20 irregular, and 20 pseudowords) and matched for frequency and length. The score in this task consists of the number of items read correctly. Norms for Brazilian children aged from 6 to 12 years and evidence of validity are available for this instrument.^29^^,^ 33
d) Phoneme Elision Task:^34^ This instrument provides a measure of phonemic awareness. The child is requested to delete a specific phoneme from a word and state what the resulting word is. Performance is scored based on the number of correct answers. The internal consistency of this instrument is .92 (KR – 20 formula).^34^
e) Digit Span (forward and backward):^35^ Brazilian version of the Wechsler Intelligence Scale for Children, Third Edition.^35^ The Digits Forward task primarily measures short-term phonological memory, while Digits Backward measures children’s ability to manipulate verbal information while holding it in a temporary storage (working memory). We employed the span measure in the present study.
f) Letter span: This instrument is analogous to the digit span task and measures short-term phonological memory and working memory. The task contains both forward and backward letter span conditions, each of which is scored on a scale from 2 to 9. The instrument was developed by the authors and showed internal consistency (Cronbach’s alpha) of .66.^36^
g) Corsi Block-Tapping Task:^37^ This task assesses visuospatial short-term and working memory. Total scores were calculated by summing the number of sequences reproduced correctly in the forward and backward trials.
h) Five Digit Test:^38^ A multilingual instrument for assessing EF and attention. The first two parts measure automatic attention and speed processing. Participants are instructed to read Arabic digits (up to five) and count randomly presented star sets (up to five). The last two subtests measure controlled attention and executive attention with inhibition and set-shifting tasks. In the inhibition task (choosing), the child counts the number of Arabic digits instead of reading them. In the set-shifting condition (switching), the child counts the number of Arabic digits in most trials, switching to reading the digits when a frame surrounds the stimulus set. Accuracy and response time are registered. Evidence of validity and reliability for Brazilian adults is available.^39^
i) Phonemic and Semantic Verbal Fluency:^40^^,^ 41 This task is part of the Brief Neuropsychological Battery for Brazilian Children (NEUPSILIN-INF). In the phonemic fluency task, children must generate as many words as possible beginning with the letter M within 60 seconds. On the semantic fluency task, they must generate as many animal names as possible, also within 60 seconds. Each task is scored based on the number of correct words produced within the time limit. Evidence of validity and reliability is available for this task for Brazilian children.^41^
j) Contingency Naming Task (CNT):^42^ This is an experimental task adapted from a study by Van der Sluis et al.,^42^ which measures rapid automatized naming (RAN)^43^ and EF. In the first three subtests, participants are instructed to name as fast as possible a set of letters (D, A, O, S), numbers (1, 2, 3, 4), and geometric figures (square, circle, triangle, and diamond). The last two subtests assess inhibitory control and set-shifting. The inhibition subtest uses the same geometric figures as the RAN subtest, but a smaller additional figure is placed in the center, and children are instructed to name the smaller figure (e.g., a triangle inside a square); that is, they have to inhibit the larger, more prepotent figures in favor of the smaller, less noticeable figure. In the set-shifting task, smaller figures are presented in the center of larger figures, and the stimulus color determines which figure has to be named: when the stimulus is black, the figure placed in the center has to be named; when the stimulus is red, the surrounding figure has to be named. Accuracy and response time are registered.^42^

### Data analysis

We performed descriptive analyses and examined the dependent variables to detect group differences to control for in further analyses. We conducted Multivariate Analysis of Variance (MANOVA), controlling for age and nonverbal reasoning, to compare performances on the neuropsychological tasks between the groups, employing the Bonferroni correction for multiple comparisons. Analyses were run in the SPSS software v. 20.0 at a significance level of 5%.

## RESULTS

A *t*-test for independent samples showed differences between cities (Porto Alegre vs. Belo Horizonte) in both reading and phonological processing. These differences may be related to the schooling process.^44^ Children from Porto Alegre had better performance on phoneme elision, *t*(204) = 4.37, *p* < .001, whereas children from Belo Horizonte were faster in RAN of letters and figures, *t*(213) = 3.58, *p* < .001 and *t*(213) = 3.39, *p* = .001, respectively. Also, in RAN of figures, children from Belo Horizonte made fewer errors than those from Porto Alegre, *t*(213) = 2.28, *p* = .05. There were no significant differences between cities in phonological short-term memory or working memory, nor in reading of regular words, irregular words, or pseudowords.

The main objective of the present study was to compare the neuropsychological performances of the ADHD symptoms group and the RD group. The RD group had significantly lower scores on the digit span forward compared to the ADHD symptoms group (phonological short-term memory) [*F*(2,168) = 3.608, *p* = .029, *Wilk’s* Λ = .865, η^2^ = .03]. They performed worse than the other groups (controls and ADHD symptoms) in phonemic elision (phonological awareness) [*F*(2,168) = 11.309, *p* < .001, *Wilk’s* Λ = .865, η^2^ = .11]; orthographic verbal fluency (lexical access, verbal memory, oral and written language, and “cool” EF such as inhibitory control) [*F*(2,210) = 9.088, *p* < .001, *Wilk’s* Λ = .752 , η^2^ = .08]; figure RAN (more errors) (speed of lexical access and phonological processing skills) [*F*(2,210) = 5.940, *p* = .003, *Wilk’s* Λ = 0.752, η^2^ = .05]; and response time – inhibitory component (processing speed and inhibitory control) [*F*(2,210) = 5.518, *p* = .005, *Wilk’s* Λ = .792, η^2^ = .10] (Table 2).

**Table 2:** MANOVA results for the neuropsychological variables, controlling for age and nonverbal IQ.

	Control group (M ± SD) (N = 159)	ADHD symptoms group (M ± SD) (N = 20)	RD group (M ± SD) (N = 37)	F	p
Phonemic elision (correct responses)	23.07 ± 4.65^a^	23.13 ± 4.65^a^	19.12 ± 4.92^b^	11.309	< .001**
Letter span - forward order	5.5 ± 1.10	5.43 ± 1.04	5.00 ± 1.10	2.629	.075
Letter span - backward order	5.37 ± 1.18	5.05 ± 1.00	4.99 ± 1.11	1.590	.207
Digit span - forward order	6.70 ± 1.31^a,b^	7.30 ± 0.96^a^	6.32 ± 1.07^b^	3.608	.029*
Digit span - backward order	3.74 ± 1.22	3.37 ± .81	3.58 ± 1.31	1.143	.321
Corsi Blocks - forward order	35.26 ± 11.65	37.82 ± 11.57	32.28 ± 12.29	1.379	.255
Corsi Blocks - backward order	26.45 ± 12.32	29.77 ± 13.62	26.56 ± 10.62	.504	.60
Orthographic verbal fluency (correct words)	7.88 ± 3.06^a^	8.20 ± 2.46^a^	5.68 ± 2.72^b^	9.088	< .001**
Semantic verbal fluency (correct words)	13.71 ± 3.56^b^	12.42 ± 2.73^a,b^	11.93 ± 4.22^a^	7.256	.001**
FDT - reading - errors	.069 ± 0.30	.096 ± 0.44	.084 ± 0.27	.088	.916
FDT - reading - RT (ms)	29784.28 ± 5651.29^b^	30684.01 ± 4808.81^a,b^	32637.81 ± 5477.81^a^	4.095	.018*
FDT - counting - errors	.47 ± 1.02^b^	.65 ± 1.18^a,b^	1.14 ± 2.98^a^	2.765	.065*
FDT - counting - RT (ms)	41766.76 ± 8621.66^b^	43197.92 ± 10282.93^a,b^	45966.71 ± 14481.08^a^	2.867	.059
FDT - choosing - errors	2.46 ± 2.65^b^	4.23 ± 4.96^a,b^	3.69 ± 4.32^a^	4.113	.018*
FDT - choosing - RT (ms)	68833.25 ± 14400.80	73480.98 ± 15313.97	72482.35 ± 14138.48	1.766	.174
FDT - switching - errors	2.98 ± 2.89^b^	4.71 ± 6.10^a,b^	4.77 ± 5.78^a^	4.417	.013*
FDT - switching - RT (ms)	79662.66 ± 18461.58	80416.20 ± 27576.06	77309.51 ± 28950.41	.231	.794
CNT - letters - errors	.10 ± .40^b^	.10 ± .44^a,b^	.32 ± .62^a^	3.443	.034*
CNT - letters - RT (ms)	20869.28 ± 4355.49^b^	22294.60 ± 5079.37^a,b^	24907.25 ± 6626.67^a^	11.779	< .001**
CNT - numbers - errors	.12 ± .52	.21 ± .41	.23 ± .68	0.750	.474
CNT - numbers - RT (ms)	22736.82 ± 5666.98^b^	23512.91 ± 6089.46^a,b^	26181.44 ± 5833.22^a^	5.393	.005*
CNT - figures - errors	.69 ± 1.69^a^	.58 ± 1.12^a^	1.82 ± 2.64^b^	5.940	.003**
CNT - figures - RT (ms)	53183.96 ± 17813.67^b^	54788.92 ± 19935.23^a,b^	63989.07 ± 17717.10^a^	5.518	.005*
CNT - figures/inhibition - errors	.53 ± 1.06^a^	1.87 ± 2.61^b^	2.12 ± 3.14^b^	14.950	< .001**
CNT - figures/ inhibition - RT (ms)	55593.30 ± 16786.00^a^	59277.15 ± 20476.31^a^	71572.31 ± 24749.91^b^	11.594	< .001**
CNT - figures/switching - errors	1.77 ± 2.84^b^	2.07 ± 2.06^a,b^	3.66 ± 5.47^a^	4.673	.010*
CNT - figures/ switching - RT (ms)	80696.08 ± 97619.91	71191.08 ± 22977.80	93043.38 ± 34444.98	.488	.614

ADHD: attention deficit hyperactivity disorder. RD: Reading Disability. FDT: Five Digit Test. RT: Response Time. Ms: Milliseconds. CNT: Contingency Naming Task. + F: analysis of variance. +^a,b^: Bonferroni post-hoc analysis.

* p < .05.

** p ≤ .001.

Regarding “cool” EF (shifting, processing speed, and verbal fluency), verbal and visuospatial working memory, and phonological processing, the performance of the ADHD symptoms group did not differ from that of the control group (*p* > .05). The only measure in which the ADHD symptoms group exhibited worse performance (larger mean number of errors) than the control group was figure RAN – inhibitory component (inhibitory control) [*F*(2,210) = 11.594, *p* < .001, *Wilk’s* Λ =.752, η^2^ = .12]. Nevertheless, their performance did not differ significantly from that of the RD group, which, as described previously, also made significantly more errors than the controls (Table 2).

## DISCUSSION

This study aimed to compare performance in “cool” EF and phonological processing between children with ADHD symptoms only, children with RD only, and their typically developing peers, after controlling for age and nonverbal reasoning. As expected, the RD group showed greater impairment in “cool” EF components (orthographic verbal fluency and processing speed) and phonological processing (phoneme awareness, short-term phonological memory, and lexical access) compared to both the ADHD symptoms and control groups.^9^^,^ 45 ^,^ 25

There is currently a consensus in the literature showing consistent associations between deficits in phonological processing and RD.^6^^,^ 7 Concerning difficulties in “cool” EF, it is crucial to consider limitations of study designs when drawing causal inferences. Relationships between EF deficits and reading performance/difficulties do not imply that EF are causal factors for reading performance. Such relationships may be reciprocal, i.e., executive abilities may develop with higher reading proficiency. Furthermore, the relationship between EF and reading tends to be stronger for more complex reading skills, such as reading comprehension.^46^ For example, after controlling for commonly accepted contributors to reading comprehension (i.e., attention, decoding skills, fluency, and vocabulary), EF continued to make a significant contribution to reading comprehension, but not to word identification skills.^10^

It is noteworthy that the tasks we employed to measure EF (orthographic verbal fluency and figure RAN), in which children with RD performed worse than children with ADHD symptoms only and control children, also require language skills. The children with RD in this study differed from the other groups in verbal memory and oral and written language abilities, such as phonological awareness and lexical-semantic processing.^47^^,^ 48 Therefore, children with RD may perform poorly on these tasks due to difficulties in verbal aspects rather than executive aspects, since verbal fluency is also related to the number of words known/learned. Indeed, vocabulary is a significant predictor of reading performance (words and text) in school-age children.^49^ Hence, the difficulties in orthographic verbal fluency observed in this sample may also be associated with linguistic variables, such as vocabulary.

Additionally, as our results showed, EF deficits are not always present in children with ADHD symptoms.^17^^,^ 25 The moderate effect sizes found in other studies and the lack of universality of EF deficits among individuals with ADHD suggest that difficulties in EF are neither necessary nor sufficient to cause ADHD.^25^ This has consequences for controversies over the validity and utility of employing EF measures in the diagnosis of ADHD, especially considering that EF measures exhibit a lack of specificity in discriminating ADHD from other clinical disorders.^50^ Nonetheless, EF tests could still be useful to characterize the cognitive profile of specific individuals.^51^

Despite the fact that children with RD displayed a more prominent profile of executive deficits in the present study, we also found a shared inhibitory control deficit between RD and ADHD children on one of the tasks, even after controlling for age and nonverbal reasoning. Inhibitory control deficits are described as an important endophenotype for the study of ADHD. This important EF component may help explain difficulties in both attention regulation and hyperactive/impulsive behaviors in children.^52^^-^ 54 However, inhibitory control deficit is not unique to ADHD. Moreover, individuals with RD showed small-to-average deficits, suggesting that impaired inhibition is not likely to be at the core of RD.^55^ Furthermore, the present study also suggests that deficits in inhibitory control observed in children with RD are probably not explained by the frequent association with high levels of inattention and hyperactivity/impulsivity, as hypothesized previously,^56^ and may be present even when no comorbidity between these conditions is present.^19^

There has been speculation regarding the distinct mechanisms underlying inhibitory control deficits in ADHD and RD. It has been suggested that children with ADHD may have a more pervasive inhibitory deficit compared to typically developing children, which leads to behavioral impulsivity, whereas children with RD may have difficulties in inhibition due to impairments in rapid information processing.^19^

In conclusion, the current results corroborate the hypothesis that RD is fundamentally characterized by neuropsychological impairments, where these are less prominent in children with ADHD symptoms.^1^ This might be explained by differences in the assessment of the two groups. Children with RD were evaluated using a standardized word reading test, i.e., a direct measure of performance, even though some did not meet all the criteria for diagnosis of dyslexia. On the other hand, inattention/hyperactivity symptoms in the ADHD symptoms group were assessed through parent/guardian reports; thus, this group did not undergo a detailed diagnostic procedure. Moreover, this type of assessment may introduce some evaluation biases, as discussed below in the Limitations section. Children with ADHD and concurrent neuropsychological deficits exhibit greater functional impairment than cases without significant neuropsychological impairments, and hence are more likely to be referred for treatment.^57^ Nevertheless, children with ADHD may not display any deficits in neuropsychological functions.^11^ The evidence for a shared deficit in inhibitory control is also in line with other studies^19^^,^ 22 and suggests a potential explanatory deficit that underlies the co-occurrence of learning disorders and ADHD, as held by the multiple deficit cognitive model.^5^

### Limitations

The results presented here must be interpreted with caution since the study has some limitations. These limitations include small sample sizes, especially for the ADHD symptoms group, and a difference in sample sizes between the two cities. It is important to observe that the proportion of girls in the RD group is twice that of boys, which contradicts the literature showing equal or greater proportion of boys with RD,^2^ indicating a sample bias. Additionally, the single source of information (parents/guardians) about children’s inattention and hyperactivity/impulsivity symptoms also constitutes a bias in this study. Measuring these indicators through teachers’ reports may contribute to greater reliability in the evaluation. The MTA-SNAP-IV questionnaire also has limitations: ADHD symptoms are nonspecific and may refer to other clinical conditions, such as anxiety. Another criticism of the use of the MTA-SNAP-IV is the limited investigation of its psychometric properties. In Brazil, the accuracy of the MTA-SNAP-IV for ADHD screening in non-clinical samples has not yet been examined.^32^ Therefore, this instrument does not have robust evidence of validity in the Brazilian context and needs further psychometric investigation.^32^

We also emphasize the need to include fine measures for other EF components, especially “hot” components. Furthermore, measures of other modalities of inhibitory control, such as motor inhibition, are needed, since the inhibitory control task employed here specifically involves inhibition of verbal responses to competing visual stimuli. Additionally, the groups in our sample were formed based solely on observed reading difficulties and ADHD symptoms, not proper diagnosis of dyslexia and ADHD. This may limit comparisons with studies that included samples selected following standardized diagnostic procedures for both conditions. We also did not split the ADHD symptoms group by subtype (inattentive, hyperactive/impulsive, and combined) due to the small sample size. However, grouping children with unspecified ADHD symptoms is consistent with the current version of the Diagnostic and Statistical Manual of Mental Disorders (DSM-5),^58^ which no longer considers the subtypes as stable categories throughout development.

In conclusion, the present study indicates that phonological measures may aid the diagnosis and intervention of RD.^8^^,^ 22 On the other hand, EF tasks cannot be used as neuropsychological markers for ADHD and RD, and impairments in verbal tasks of inhibitory control with visual stimuli may not differentiate clinical groups with these conditions.^22^ However, we would like to highlight the importance of assessing executive difficulties, as well as difficulties in other neuropsychological domains, in children with RD or those with inattention/hyperactivity symptoms above those expected for their age and development. Early neuropsychological assessment and interventions for EF domains and phonological processing may help promote the adequate development of other cognitive functions, functional adaptation, and academic performance of such children.
